# Successful pancreatic duct cannulation using a novel rotatable dual-action sphincterotome at the pancreaticojejunostomy site

**DOI:** 10.1055/a-2693-8276

**Published:** 2025-09-11

**Authors:** Yuki Fujii, Kazuyuki Matsumoto, Koichiro Tsutsumi, Kosei Takagi, Kazuya Yasui, Tomokazu Fuji, Motoyuki Otsuka

**Affiliations:** 1 Department of Gastroenterology and Hepatology, Okayama University Graduate School of Medicine, Dentistry and Pharmaceutical Sciences, Okayama, Japan; 2 Department of Gastroenterological Surgery, Okayama University Graduate School of Medicine, Dentistry and Pharmaceutical Sciences, Okayama, Japan

In patients with a surgically altered anatomy, balloon-endoscopy-assisted endoscopic retrograde cholangiopancreatography (BE-ERCP) is widely used to treat pancreatobiliary diseases 1 . However, successful pancreaticojejunostomy site cannulation remains technically challenging because identifying the anastomosis and performing cannulation at a steep angle is difficult 2 3 . We present the use of a novel sphincterotome with high rotational capability and dual-action control (Engetsu; Kaneka Medix Co., Osaka, Japan) that has recently become available, enabling precise directional control of the guidewire, even in anatomically complex settings, and potentially improving the cannulation success rate ( Fig. 1 ).

**Fig. 1:**
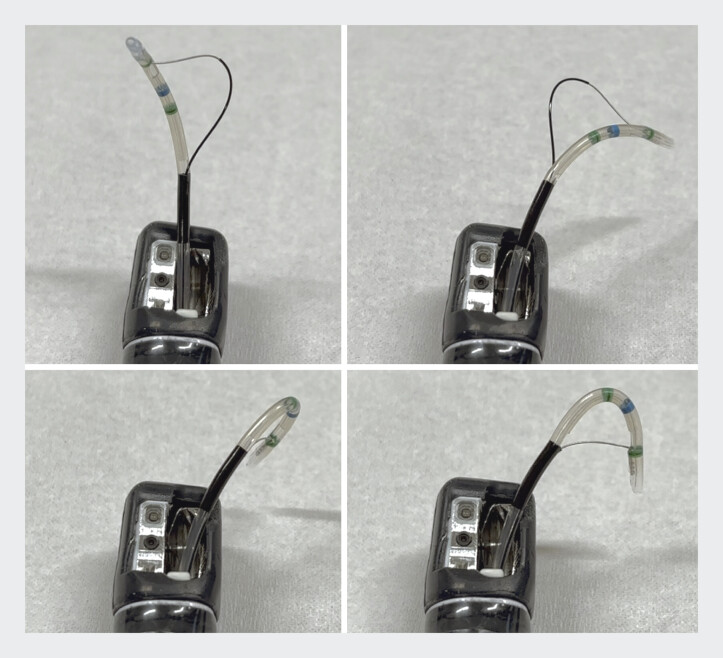
The novel sphincterotome (Engetsu; Kaneka Medix Co., Osaka, Japan) has high rotatability and a dual-action mechanism. Pulling tightens the blade, while pushing loosens it. The tip can be precisely oriented in any direction by combining these functions.

A 69-year-old man presented at our hospital with abdominal pain and melena. Contrast-enhanced computed tomography (CE-CT) revealed a large duodenal bulb ulcer with retroperitoneal perforation. Transcatheter arterial embolization was attempted for hemostasis but was unsuccessful, and emergency pancreaticoduodenectomy was performed. On postoperative day 8, CE-CT was performed due to fever. Although the “lost” pancreatic duct stent had not migrated, a pancreatic fistula was observed ( Fig. 2 ). Considering the possibility of early occlusion of the lost duct stent, BE-ERCP was performed. A balloon endoscope (EI-580BT; Fujifilm Co., Tokyo, Japan) was advanced to the pancreaticojejunostomy site. Although the lost duct stent was identified, cannulation of the pancreatic duct with a conventional catheter was unsuccessful because of sharp angulation. By utilizing the rotational capability of the novel sphincterotome and adjusting the push and pull modes, guidewire insertion into the pancreatic duct was successfully achieved using the wire-guided cannulation technique ( Fig. 3 , Video 1 ). Subsequent contrast injection showed both the leakage cavity and the pancreatic duct. After removal of the lost duct stent, a 5-Fr, 7-cm stent (Harmoray; Hanaco Medical Co., Saitama, Japan) was successfully placed into the pancreatic duct ( Fig. 4 ). Follow-up CE-CT confirmed the resolution of the pancreatic fistula, and the patient was subsequently discharged ( Fig. 5 ).

**Fig. 2:**
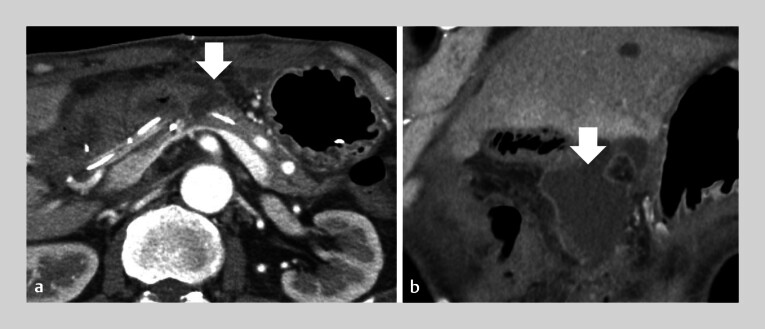
**a, b** Contrast-enhanced computed tomography (CE-CT) on postoperative day 8 shows a pancreatic fistula (arrow).

**Fig. 3:**
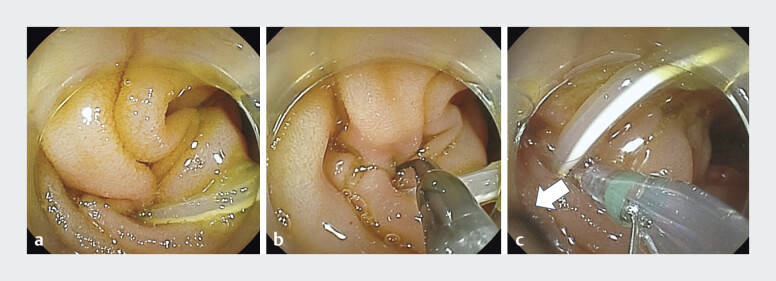
**a** A balloon endoscope is advanced to the pancreaticojejunostomy site. **b** Cannulation using a conventional catheter fails due to sharp angulation. **c** Guidewire insertion is successful with the novel sphincterotome using wire-guided cannulation. The arrow indicates the direction of the pancreatic duct.

Demonstration of successful pancreatic duct cannulation using the novel sphincterotome at the pancreaticojejunostomy site.Video 1

**Fig. 4:**
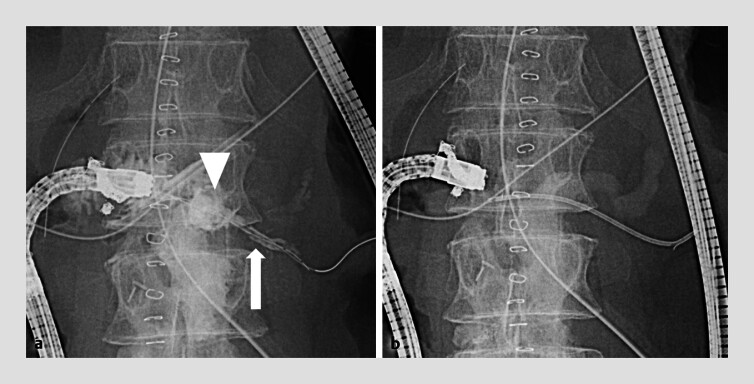
**a** Contrast reveals both the leakage cavity (arrowhead) and pancreatic duct (arrow). **b** A 5-Fr, 7-cm stent is placed into the pancreatic duct.

**Fig. 5:**
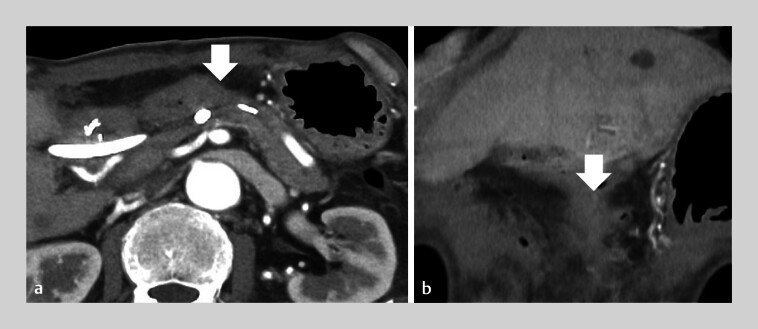
**a, b** Follow-up CE-CT confirms resolution of the pancreatic fistula (arrow).

Endoscopy_UCTN_Code_TTT_1AR_2AC

## Notes

Endoscopy E-Videos https://eref.thieme.de/e-videos
*E-Videos* is an open access online section of the journal *Endoscopy* , reporting on interesting cases and new techniques in gastroenterological endoscopy. All papers include a high-quality video and are published with a Creative Commons CC-BY license. Endoscopy E-Videos qualify for HINARI discounts and waivers and eligibility is automatically checked during the submission process. We grant 100% waivers to articles whose corresponding authors are based in Group A countries and 50% waivers to those who are based in Group B countries as classified by Research4Life (see: https://www.research4life.org/access/eligibility/ ). This section has its own submission website at https://mc.manuscriptcentral.com/e-videos .

## References

[1] Shimatani M Matsushita M Takaoka M Effective “short” double-balloon enteroscope for diagnostic and therapeutic ERCP in patients with altered gastrointestinal anatomy: a large case series Endoscopy 2009 41 849 854 19750447 10.1055/s-0029-1215108

[2] Basiliya K Veldhuijzen G Gerges C Endoscopic retrograde pancreatography-guided versus endoscopic ultrasound-guided technique for pancreatic duct cannulation in patients with pancreaticojejunostomy stenosis: a systematic literature review Endoscopy 2021 53 266 276 32544958 10.1055/a-1200-0199

[3] Calcagno P Mazzola M Forti E Endoscopic management of postoperative pancreatic fistula after pancreaticoduodenectomy: a single center retrospective analysis Surg Endosc 2025 39 4186 4194 10.1007/s00464-025-11786-2 40372448

